# Adhesive Capabilities of *Staphylococcus Aureus* and *Pseudomonas Aeruginosa* Isolated from Tears of HIV/AIDS Patients to Soft Contact Lenses

**DOI:** 10.5539/gjhs.v4n1p140

**Published:** 2012-01-01

**Authors:** Ajayi B. O., Kio F.E., Otajevwo F.D.

**Affiliations:** Dept of Optometry, University of Benin Nigeria; Dept of Microbiology & Biotechnology, Western Delta University Oghara, Nigeria

**Keywords:** HIV, Contact lenses, Adhesiveness, *Staph. aureus*, *Pseudo. aeruginosa*, Tears

## Abstract

Fifty conjunctival swab samples collected from ELISA confirmed HIV/AIDS seropositive patients who were referred to the HIV/AIDS laboratories of the University of Benin Teaching Hospital and Central Hospital both based in Benin City, Nigeria were aseptically cultured on appropriate media by standard methods. The resulting isolates/strains, after identification by standard methods, were tested for their ability to adhere to two hydrophobic non-ionic daily wear silicone hydrogel soft contact lenses (i.e. lotrafilcon B, WC 33% and polymacon, WC 38%) as well as to two hydrophilic ionic conventional extended wear silicone hydrogel soft contact lenses (i.e. methafilcon A, WC 55% and omafilcon A, WC 60%) by the adhesiveness/slime production modified vortex/Robin device method. Evidence of adhesiveness/slime production was indicated by presence of a visible stained film lining the surface of the contact lens which was measured and recorded as strong or weak according to the density of the adhered bacterial film. Fourteen (28.0%) *Staphylococcus aureus* strains and 10 (20.0%) *Pseudomonas aeruginosa* strains were obtained among other organisms. *Staphylococcus aureus* strains adhered in decreasing order to lotrafilcon B (55.4 ± 4.7), polymacon (46.4 ± 8.4), methfilcon A (46.4 ± 8.4) and omafilcon A (25.0 ± 6.4) with no significant difference in adhesive strengths of individual strains (P > 0.05). *Pseudomonas aeruginosa* strains also recorded decreasing adhesive strengths to lotrafilcon B (37.5 ± 8.2), polymacon (28.6 ± 6.3), methafilcon A (26.8 ± 5.5) and omafilcon A (23.2 ± 5.5) also with no significant difference in adhesive strengths of individual strains (P > 0.05). Attachment strengths of *Staph. aureus* strains to all four contact lenses were higher than those of *Pseudomonas aeruginosa* strains. Both organisms adhered most to hydrophobic lotrafilcon B and least to hydrophilic omafilcon A. This invitro adhesion studies revealed that daily wear silicone hydrogel low water content, non-ionic contact lenses are more prone to bacterial adhesion than the conventional extended wear hydrogel high water content, ionic contact lenses and hence, there is more risk of microbial adhesion to the former compared to the latter. Other implications are highlighted.

## 1. Introduction

Contact lenses are the smallest, the least visible devices for correcting refractive error of the eyes. It is a shell-like, bowl shaped glass or plastic that rests on the eye (Mandel, 1981). Contact lens uses range from cosmetic to functional e.g. sports (Poster, 1972). Contact lens fitting is indicated in the management of severe ocular pathology, keratoconus and monocular aphakia although fitting in the presence of active pathology should never be undertaken. Studies have shown that 96% of patients fitted with contact lenses find them a complete success in terms of improved vision (Anon, 1990).

There are two categories of soft contact lenses and they are hydrophilic and hydrophobic types. Hydrophilic lenses allow the passage of water molecules, gas molecules being transported with the water molecules. Soft hydrophilic lenses are divided into those with low water content and those with high water content. Silicon hydrophilic soft contact lenses are a new generation of supra permeable contact lenses that can transmit unprecedented amounts of oxygen to the cornea. It represents a breakthrough over traditional hydrogel soft contact lenses because silicon allows so much oxygen through the lens. Silicon hydrogel soft contact lenses are made from hydrogel polymers.

The conventional soft lenses are based on polyhydroxyl-ethylmetacrylate (P-HEMA). The lens materials are co-polymers of HEMA and other hydrophilic monomers such as N-vinyl pyrrolidine (NVP) and metacrylates that possess a wide range of water content. The water content is usually above 38% which contributes to the softness and comfort of these lenses. Less than 50% water content is considered to be low water content lenses and greater than 50% water are high water content lenses.

Material surfaces can be considered hydrophobic if the water contact angle is higher than 50°. Lotrafilcon B (WC 33%) and polymacon (38%) are low water content hydrophobic silicon hydrogel contact lenses while methafilcon A (WC 55%) and omafilcon A (WC 60%) are high water content hydrophilic hydrogel lenses.

Studies have suggested that hydrophobic surfaces are more prone to pathogenic adhesion than hydrophilic ones. Silicon hydrogel contact lens is more prone to bacterial adhesion and this is attributed to the fact that silicon hydrogel lenses have a surface hydrophobicity higher than that of the conventional hydrogel lenses. Holden (2002) disproved this by stating that the adhesion of bacteria to silicon hydrogel contact lenses are as a result of the inherent property of the polymer or the unoxidized surface after treatment.

Cellular adhesion is the binding of a cell to another cell or to a surface or matrix. Bacterial adhesion is the process by which bacteria stick to the surface of host cells. Cellular adhesion is regulated by specific cell adhesion molecules that interact with other molecules. Pilli or fimbriae of gram negative bacteria such as *Pseudomonas aeruginosa* play an important role in adhesion to cell surface. Microorganisms are considered to play a role in the aetiology of certain corneal infiltrative events observed during soft contact lens wear (Padmaja *et al.*, 2000). Buehler *et al*. (1992) reported that adhesion of bacteria notably *Staphylococcus* strains and *Pseudomonas aeruginosa* to contact lenses is considered a primary risk factor.

The mechanism used by bacteria to attach to the contact lens surface is poorly understood. Bacteria are thought to attach to a contact lens by interaction of the outer lipoprotein layer with the lens. Once placed in the eye, the contact lens undergoes a profound change in its surface properties. Species of bacteria are however, believed to bind specifically to the carbohydrate residues of this protein including *P. aeruginosa*, *Escherichia coli* etc. *P. aeruginosa* is known to secrete an anionic polysaccharide biofilm matrix on the lens surface in which the organisms are known to metabolize and reproduce. Borazjani *et al*. (2004) however, found no marked differences in the adhesion of *P. aeruginosa* to worn and unworn silicon hydrogel lenses thus suggesting that these lens surface properties were not affected by 6-7days extended wear and thus by the presence of tear film molecules.

Microbial contamination of the lens surface is the main problem associated with contact lenses wear. Although the estimated risk of the incidence of silicon hydrogel lens associated keratitis is one in 15,800 patients yearly, it is 30 times lower than for conventional hydrogel types (Lam *et al.*, 2002; Lee *et al.*, 2003). This was further elaborated in a study where the frequency of negative cultures was reported to be significantly greater during asymptomatic lens wear in comparison to symptomatic corneal infiltration. Conversely, the frequency of isolation of gram positive bacteria, gram negative bacteria and fungi was significantly greater in symptomatic corneal infiltration than asymptomatic lens wear suggesting that the use of contact lens can pose a threat in terms of bacterial invasion of the ocular tissue.

Factors that play important roles in the adhesion process of bacteria to contact lenses include: surface hydrophobicity/net surface charge, host receptor interaction and binding molecules present on the bacterial cells. Bacterial adherence to epithelial surface occurs due to molecular interactions between bacterial surface proteins and protein receptors on the cell surfaces. Surface hydrophobicity of the contact lens has been found to enhance bacterial adhesion. Bacterial host cells usually have net negative surface charge and therefore repulsive electrostatic forces.

Fujikawa *et al*. (1985) showed that when patients contract HIV, the virus can infect nearly every ocular tissue as well as the tears. The tear gland surface, like every other ocular surface is colonized by microbial agents which are mainly commensals such as *Staphylococcus epidermidis, Staphylococcus aureus, Corynebacterium* spp and *Propionibacterium acnes*. Bacteria commonly isolated from ocular infections include gram positive cocci, *Pseudomonas aeruginosa* among others (Shivitz, 1987). The ability of the bacteria to attach to the lens may depend on the type of lens material, immediate environmental conditions or the bacteria themselves.

Human T-cell viruses (HTLV-III) have been found in tears thus indicating the presence of free virus in tears (Fujikawa *et al.*, 1986). Retroviral patients in their immune compromised state would have a wider range of bacterial organisms present in their eyes. These organisms include *Staphylococcus aureus* and *Pseudomonas aeruginosa* among others (Yasuyuki and Ben, 2005). *Staphylococcal* and *Pseudomonas* organisms are opportunistic pathogens in humans which infect the eyes through contaminated fingers/contact lenses.

Silicone-hydrogel soft contact lenses have been found to be more prone to bacterial adhesion than conventional hydrogel soft contact lenses and this is attributable to the hydrophobic nature of the lens. Laurent *et al*. (2002) reported that the extent of bacterial binding was found to range in increasing order from hydrogel to fluorine polyhydroxyl-ethylmetacrylate (PMMA), to hydrophilic acrylic, to heparinized PMMA, to silicone polymer contact lens types.

Mowrey-Mckee *et al*. (1992) carried out a study to determine the relative adhesion of bacteria to HEMA-type contact lenses and an extended wear silicon hydrogel contact lens of high oxygen permeability. They reported that adhesion of *P. aeruginosa* to a hydrogel contact lens does not appear to differ appreciably between the HEMA-type etafilcon and the high silicon hydrogel balafilcon A lens. The ability of *Staphylococcus. epidermidis* 9112 to adhere to the hydrophobic silicone hydrogel lotrafilcon A and balafilcon A was also greater than adhesion to the hydrophilic etafilcon A type thus establishing that hydrophobic silicone lenses are more prone to bacterial adhesion.

*Pseudomonas aeruginosa* has been shown to adhere strongly to contact lens of low water content than those of high water content. Hart *et al*. (1993) carried out an invitro quantitative study of the adhesion of a *Staphylococcus aureus* strain to two types of disposable contact lenses of ionic and non-ionic water content and reported that the ionic water content lenses were more prone to *Staphylococcus aureus* adhesion than the non-ionic water content lenses. It has been reported that daily wear of soft contact lenses significantly increased the binding of *Pseudomonas aeruginosa* to exfoliated epithelial cells and this binding is inversely proportional to the oxygen transmissibility of the contact lens (Butrus *et al.*, 1997).

The effect of continuous wear on physico-chemical surface properties of silicone hydrogel lenses and their susceptibility to bacterial adhesion was studied of which volunteers were made to wear two pairs of either lotrafilcon A or balafilcon A contact lenses. The first pair was worn continuously for a week and the second pair for 4 weeks. One lens of each pair was used for surface characterization and the other one for bacterial adhesion experiments. Lens surfaces were characterized by examination of their wettability, roughness, elemental composition and proteins attached to their surfaces. Results showed that bacteria adhered in lower numbers and less tenaciously to worn lenses except *Staph. aureus* which adhered in higher numbers to worn balafilcon A lenses (Bos *et al.*, 1999).

Robert *et al*. (2002) also carried out a study to determine if *Pseudomonas aeruginosa* has the ability to adhere preferentially to unused contact lenses made from different group polymers. They reported that the polymer material (used to construct the contact lenses) may influence subsequent bacterial adhesion and hence, concluded that contact lenses made from non-ionic polymers with low water content may carry higher risks of bacterial contamination.

HIV/AIDS is a disease condition that can affect every ocular tissue as the tear gland and hence, the tears. Contact lens wear by sufferers of this condition therefore may increase the ability of microbial cells to adhere to the cornea leading to keratitis especially if not properly handled during wearing and storage. Extended wear of contact lenses will therefore save sufferers the trouble of constantly removing and storing and this is why extended wear lenses are better than daily wear contact lenses. The risks associated however, with silicone hydrogel lenses on HIV/AIDS patients in terms of microbial contamination have not been fully investigated. This study is therefore aimed at determining the relative adhesion capacities of *Pseudomonas aeruginosa* and *Staphylococcus aureus* isolated from tears of HIV/AIDS patients to the recently manufactured commercially available hydrogel soft contact lenses with the following objectives: 1. Determine the measure of adhesiveness of *Staphylococcus aureus* strains to the selected hydrogel contact lenses. 2. Determine the measure of adhesiveness of *Pseudomonas aeruginosa* strains to the selected hydrogel contact lenses.

## 2. Materials and Methods

Eye (conjunctival) swabs were carefully collected from 50 ELISA test – confirmed seropositive HIV/AIDS patients who visited the University of Benin Teaching Hospital (UBTH) and Central Hospitals both based in Benin City, Nigeria. To obtain conjunctival swabs, the lower lids of the eye were lowered down gently and the palpebral conjunctiva/culdesac was swabbed with sterile swab sticks. Specimens were collected with informed consent from the ethical committees of the hospitals involved through correspondences between the Optometry department, University of Benin and the hospitals.

Conjunctival swabs were aseptically cultured on sterile MacConKey agar, Blood agar and Mannitol Salt agar plates and incubated aerobically at 37°C for 24hours. Pure isolates/strains were obtained and stocked on nutrient agar slants. Pure isolates were then identified culturally, morphologically, biochemically and by sugar fermentation tests according to schemes provided by Cowan and Steel (1993) and Cullimoore (2000).

All circular, white – yellowish, raised entire colonies, gram positive, coagulase positive, glucose positive and mannitol positive colonies/strains (characteristic of *Staphylococcus aureus*) and short gram negative rods in singles, citrate positive, oxidase positive, gray – greenish pigmented colonies/strains (characteristic of *Pseudomonas aeruginosa*) were used for further studies. The *Staphylococcus aureus* and *Pseudomonas aeruginosa* isolates and strains were then subjected to the adhesive capability and slime production assay.

## 3. Adhesive Capability/Slime Production Assay

The hydrogel lenses used in this study were daily wear silicone hydrogel, non – ionic lotrafilcon B (WC 33%), extended wear hydrophilic, ionic omafilcon A (WC 60%), daily wear hydrophobic silicone hydrogel, non-ionic polymacon (WC 38%) and hydrophilic daily wear ionic methafilcon A (WC 55%).

The adhesiveness/slime production assay method used was the modified Vortex/Robin device described by Bertoluzza *et al*. (2004). Each isolate/strain was subcultured aseptically (from their slant stock cultures) into sterile nutrient broth and incubated aerobically at 37°C for 24 hours. Three milliliters (3ml) of turbid broth culture of each organism was inoculated into a set of 5ml tryptone soya broth in sterile test tubes already containing the different contact lenses listed. The mouths of all inoculated test tubes were bunsen flamed, sealed properly and incubated at 37°C for 24 hours.

The content of each tube was carefully aspirated leaving the contact lens inside each tube. The contact lens (inside each tube) was then stained with safranin solution for 30 minutes. The contact lens was then taken out of each tube, placed with the convex side up on a blotting paper and then viewed under X 10 objective of a compound microscope.

Evidence of adhesiveness/slime production was indicated by presence of a visible stained film lining the surface of the contact lens and this was measured and recorded as weak or strong according to the density of the adhered bacterial film.

## 4. Data Analysis

Data obtained were analyzed using the statistical package for social scientists (SPSS) versions 16.0 and 17.0. One way analysis of variance and Duncan’s multi-sample test was used to compare the adhesiveness of each contact lens type and for each strain. All analyses were performed at 95% confidence level.

## 5. Results

Out of the 50 samples processed, 14 (28.0%) *Staphylococcus aureus* strains and 10 (20.0%) *Pseudomonas aeruginosa* strains were obtained among other organisms. *Staph. aureus* and *Pseudomonas aeruginosa* were selected for the study due to their high occurrence in ocular infections and their apparently high resistance to most commonly used eye drops and drugs. *Pseudomonas aeruginosa* for instance, has been severally reported to thrive in commonly used disinfectants. Contact lens solutions also have disinfecting effects.

*Staphylococcus aureus* strains adhered in decreasing order to lotrafilcon B (55.36 ± 4.7), polymacon (46.4 ± 8.4), methafilcon A (46.4 ± 8.4) and omafilcon A (25.0 ± 6.4). There was no significant difference in the individual adhesion strength values for each strain to all four contact lenses sampled (P > 0.05) Table 1. Hence, by implication, *Staph.aureus* strains adhered most to lotrafilcon B contact lens and least to omafilcon A (Table 1). Whereas the attachment of the strains to lotrafilcon B was strong, that to omafilcon A was weak. Attachment strengths to polymacon and methafilcon however, were either weak or strong.

**Table 1:** Measure of Adhesiveness of 14 strains of *Staphylococcus aureus* on the four sampled contact lenses

Strain No	LotrafilconB	Polymacon	Methafilcon A	Omafilcon A
	(WC33%)	(WC 38%)	(WC 55%)	(WC 60%)
	Daily wear	Daily wear	Conventional	Conventional
SA_1_	50	50	25	50
SA_2_	75	50	25	0
SA_3_	50	50	50	25
SA_4_	50	75	75	0
SA_5_	75	25	50	25
SA_6_	50	75	25	50
SA_7_	75	25	50	0
SA_8_	25	75	50	0
SA_9_	25	50	50	25
SA_10_	50	75	75	75
SA_11_	75	75	50	50
SA_12_	50	50	25	25
SA_13_	50	25	50	25
SA_14_	75	25	50	0
Mean ± S.E	55.4 ± 4.7	46.4 ± 8.4	46.4 ± 8.4	25.0 ± 6.4
P-value	P > 0.05	P > 0.05	P > 0.05	P > 0.05

SA = *Staphylococcus aureus,* 0 = No attachment, 25 = Weak attachment, 50 = Strong attachment, 75 = Very strong attachment (Bertoluzza *et al*., 2004)

As in the case of *Staphylococcus aureus*, *Pseudomonas aeruginosa* strains recorded decreasing attachment strengths from lotrafilcon B (37.5 ± 8.2), polymacon (28.6 ± 6.3), methafilcon A (26.8 ± 5.5) and omafilcon A (23.2 ± 5.5). There was also no statistical significant difference in the individual strain attachment strengths to the four sampled lenses (P > 0.05) Table 2. The attachment strengths of *Pseudomonas aeruginosa* strains however to the sampled lenses were obviously much lower compared to those of *Staph. aureus* strains to the same lenses.

**Table 2:** Measure of Adhesiveness of 10 strains of *Pseudomonas aeruginosa* on the four sampled contact lenses

Strain No	LotrafilconB	Polymacon	Methafilcon A	Omafilcon A
	(WC33%)	(WC 38%)	(WC 55%)	(WC 60%)
	Daily wear	Daily wear	Conventional	Conventional
PA_1_	25	50	50	25
PA_2_	75	25	50	50
PA_3_	50	25	50	25
PA_4_	75	25	25	50
PA_5_	50	50	25	50
PA_6_	25	75	25	25
PA_7_	25	50	50	0
PA_8_	75	25	50	25
PA_9_	50	50	25	50
PA_10_	75	25	25	25
Mean ± S.E	37.5 ± 8.2	28.6 ± 6.3	26.8 ± 5.5	23.2 ± 5.5
P-value	P > 0.05	P > 0.05	P > 0.05	P > 0.05

PA = *Pseudomonas aeruginosa,* 0 = No Attachment, 25 = Weak Attachment, 50 = Strong Attachment 75 = Very strong attachment (Bertoluzza *et al*., 2004)

Whereas *Pseudomonas aeruginosa* strains attached most to lotrafilcon B, they attached least to omafilcon A. Attachment strength of *P. aeruginosa* strains to all sampled lenses was weak (Table 2). Both *Staph. aureus* and *P. aeruginosa* strains attached highest to lotrafilcon B and lowest to omafilcon A.

## 6. Discussion

*Staphylococcus aureus* and *Pseudomonas aeruginosa* were selected and used for this study because they are the most occurring isolates present in most ocular infections (Henriques *et al.*, 2005). In a study carried out by Reichert and Stern (1984), *Staph. aureus*, *Streptococcus pneumoniae* and *Pseudomonas aeruginosa* were found to adhere to corneal epithelium significantly.

Lotrafilcon B (non-ionic) lens having water content of 33% and polymacon (non-ionic) having water content of 38% represent hydrophobic daily wear silicone hydrogel soft contact lenses while omafilcon (ionic) and methafilcon A having water content of 60% and 55% respectively, represent hydrophilic conventional extended wear silicone hydrogel soft contact lenses. The incorporation of silicone into a hydrogel polymer gives the advantage of high oxygen transmissibility, but the disadvantage of decreased hydrophilicity (Tighe, 1999). To render the surface hydrophilic, techniques incorporating plasma into the surface of the lens have been developed.

The two daily wear silicone hydrogel lenses have a surface hydrophobicity higher than that of conventional extended wear silicone hydrogel soft contact lenses. These differences in surface hydrophobicity may explain the differences found in bacterial adhesion. Many studies have suggested that hydrophobic surfaces are more prone to pathogens adhesion than hydrophilic ones (Gomez-Suarez *et al.*, 1999; Doyle, 2000). Beattie *et al*. (2003) studied *Acanthamoeba* attachment to a silicone hydrogel lens (balafilcon A) and conventional hydrogel contact lenses and concluded that balafilcon A is more prone to bacterial adhesion. They suggested that the high levels of attachment found in silicone hydrogel lenses may be the result of the inherent property of the polymer.

Our results showed that *Staph. aureus* strains recorded strong adhesion to both hydrophobic daily wear soft contact lenses (i.e. lotrafilcon B and polymacon) of 55.4 ± 4.7 and 46.4 ± 8.4 respectively. Conversely, adhesion strengths to the two hydrophilic conventional extended wear contact lenses (methafilcon A and omafilcon A) by *Staph. aureus* strains were 46.4 ± 8.4 and 25.0 ± 6.4 respectively and this was near weak on average.

The adhesiveness of individual strains to all four lenses was not significantly different from each other (P > 0.05). This report is similar to the finding of Grosvenor (2002) which stated that hydrophobic lenses cause higher adhesion because of the formation of biofilms with inherent properties of the polymer of the lens by a biofilm positive strain.

Lotrafilcon B has the lowest water content compared to the others. Omafilcon A has the highest water content. This suggests that contact lenses of low water content are more prone to bacterial adhesion. This explains the finding in this study in which *Staph. aureus* strains adhered greatest to lotrafilcon B followed by polymacon (both of which have low water content and are hydrophobic). The effect of low water content on bacterial adhesiveness to contact lenses was demonstrated by an invitro study carried out by Butrus *et al*. (1997) to determine increased *Pseudomonas aeruginosa* adhesion following five minutes air drying of etafilcon A soft contact lens and reported an increased bacterial adhesion afterwards. They concluded that soft contact lens recorded raised drying results in increased bacterial adhesion.

*Pseudomonas aeruginosa* strains, like *Staph. aureus* strains reported the highest adhesion strengths to lotrafilcon B followed by polymacon, methafilcon A and omafilcon A. Unlike *Staph. aureus*, the strains of *P. aeruginosa* recorded reduced adhesion strengths of 37.5 ± 8.2, 28.6 ± 6.3, 26.8 ± 5.5 and 23.2 ± 5.5 to lotrafilcon B, polymacon, methafilcon A and omafilcon A respectively. *Staph. aureus* strains and *P. aeruginosa* strains recorded low adhesion to the high water containing (hydrophilic) conventional extended wear lenses of methafilcon A and omafilcon A. This is supported by Grosvenor (2002) who stated that the gas permeability of a hydrogel lens increases exponentially with the water content thereby suggesting that hydrophilic contact lenses will provide better oxygen supply to the cornea and hence possess lower risk of bacterial adhesion This, somewhat differs from the finding of Willcox *et al*. (2001) who reported an increased capability of *P. aeruginosa* to adhere to silicone-hydrogel balafilcon A when compared with the adhesion to conventional hydrogels. Conversely, Borazjani *et al*. (2004) found no significant differences between the adhesion of *P. aeruginosa* to silicon-hydrogel balafilcon A and etafilcon A. These contradictory results may be the result of the different bacterial strains used as well as varying growth conditions employed.

Several authors have reported that the extent of *P. aeruginosa* adherence is strain-dependent and influenced by growth stage and media (Willcox *et al.*, 2001; Thuruthyil *et al.*, 2001; Bruinsma *et al.*, 2002; Cowell *et al.*, 1999). However, studies have shown that deposits accumulation can increase with the length of wear of high water content disposable lenses (Maissa and Franklin, 1998).

In a study carried out to compare the adhesion patterns of three strains of *P. aeruginosa*, it was found that the number of adhered cells of *P. aeruginosa* to etafilcon A was significantly higher than that of *Staphylococcus epidermidis* thus re-enforcing the idea that the hydrophobic silicone lenses are more prone to bacterial adhesion.

Means of adhesion strengths of both organisms to all four contact lenses also showed decreasing adhesion levels from lotrafilcon B, polymacon, methafilcon A to omafilcon B although *Staph. aureus* strains clearly showed much greater adhesion strengths. Based on data obtained, it could be speculated that there are obvious risks associated with hydrophobic silicone hydrogel daily wear contact lenses as compared with the conventional extended wear types in terms of higher microbial adhesion.

This study may provide an indication of the likely transference of bacterial organisms from the wearer’s fingers to the contact lenses surfaces. Borazjani *et al*. (2004) however, found no marked differences in the adhesion of *P. aeruginosa* to worn and unworn silicon-hydrogel lenses. Our study is however limited by a small sample size among others. A much larger sample size of contact lens users would show a clearer picture of the extend of bacterial adherence to contact lenses and its far reaching health implications.

## 7. Conclusion

*Staphylococcus aureus* strains adhered in decreasing order to lotrafilcon B (55.4 ± 4.7), polymacon (46.4 ± 8.4), methafilcon A (46.4 ± 8.4) and omafilcon (25.0 ± 6.4). Whereas the first two contact lens types are hydrophobic, the last two are hydrophilic. *Staph. aureus* strains therefore adhered most to the hydrophobic lenses. *Pseudomonas aeruginosa* strains also adhered (but with much lower adhesive strengths) in decreasing order to lotrafilcon B (37.5 ± 8.2), polymacon (28.6 ± 6.3), methafilcon A (26.8 ± 5.5) and omafilcon (23.2 ± 5.5).

Both *Staph. aureus* and *P. aeruginosa* strains attached highest to hydrophobic lotrafilcon B and lowest to hydrophilic omafilcon lens. Hence, *Staph. aureus* strains exhibited greater adhesion to daily wear hydrophobic, non-ionic silicone hydrogel lotrafilcon B with water content of 33% while the least adherence was to extended wear hydrophilic ionic silicone hydrogel omafilcon lens. *Pseudomonas aeruginosa* strains exhibited the same but with reduced adhesive strengths.

This invitro adhesion studies revealed that daily wear hydrogel low water content, non-ionic contact lenses are more prone to bacterial adhesion than the conventional extended wear hydrogel high water content, ionic contact lenses and hence, there is more risk of microbial adhesion with the former compared to the latter.

Patients presenting with ophthalmic manifestation of HIV/AIDS opportunistic infections should be discouraged from using hydrophobic ionic daily wear soft contact lenses as it may supply an inoculum of organism on prolonged contact with the cornea thus increasing the risk of cornea infiltration.

The use and advantages of conventional extended wear silicone hydrogel lenses over the daily wear types should be stressed and encouraged by practitioners in the field.
